# Comorbidities and HCV coinfection in the management of HIV+ patients: evidence from the Italian clinical practice

**DOI:** 10.1186/s13561-020-00284-x

**Published:** 2020-08-29

**Authors:** Elisabetta Garagiola, Emanuela Foglia, Lucrezia Ferrario, Paola Meraviglia, Alessandro Tebini, Barbara Menzaghi, Chiara Atzori, Giuliano Rizzardini, Teresa Bini, Antonella D’Arminio Monforte, Davide Croce

**Affiliations:** 1 Centre for Research on Health Economics, Social and Health Care Management, LIUC–Università Cattaneo, Castellanza, Italy; 2 Fatebenefratelli Sacco Hospital, Milan, Italy; 3 Valle Olona Hospital, Busto Arsizio, Italy; 4 School of Clinical Medicine, Faculty of Health Science, University of the Witwatersrand, Johannesburg, South Africa; 5 Santi Paolo e Carlo Hospital, Milan, Italy; 6 School of Public Health, Faculty of Health Sciences, University of the Witwatersrand, Johannesburg, South Africa

**Keywords:** HIV +, HIV/HCV, Comorbidities, Management, Economic evaluation

## Abstract

**Background:**

Since HIV+ treatment has become more effective, the average age of people living with HIV (PLWHIV) has increased, and consequently the incidence of developing comorbidities, making the clinical and economic management of HIV+ patients more complex. Limited literature exists regarding the management of comorbidities costs. This study is aimed at defining and comparing the total annual costs of comorbidities, in an Italian cohort of HIV and HIV/HCV patients, from the National Healthcare Service perspective. The authors hypothesised that there are higher costs, for patients with multiple comorbidities, and a greater consumption of resources for HIV/HCV co-infected patients versus HIV mono-infected patients.

**Methods:**

An observational retrospective multi-centre health-economics study, enrolling HIV+ and HIV/HCV consecutive patients with at least one comorbidity, was conducted. The consecutive cases, provided by three Italian infectious diseases centres, were related to the year 2016. The enrolled patients were on a stable antiviral therapy for at least six months. Demographic and clinical information was recorded. Costs related to HIV and HCV therapies, other treatments, medical examinations, hospitalizations and outpatient visits were evaluated. Data from mono-infected and co-infected groups of patients were compared, and the statistical analysis was performed by t-tests, chi-square and ANOVA. A sub-analysis excluding HCV therapy costs, was also conducted. The hierarchical sequential linear regression model was used to explore the determinants of costs, considering the investigated comorbidities. All analyses were conducted with a significant level of 0.05.

**Results:**

A total of 676 patients, 82% male, mean age 52, were identified and divided into groups (338 mono-infected HIV+ and 338 co-infected HIV/HCV patients), comparable in terms of age, gender, and demographic characteristics. A trend towards higher annual costs, for patients with multiple comorbidities was observed in HIV mono-infected patients (respectively € 8272.18 for patients without comorbidities and € 12,532.49 for patients with three or more comorbidities, *p*-value: 0.001). Excluding anti-HCV therapies costs, HIV/HCV co-infected patients generally required more resources, with statistically significant differences related to cardiovascular events (€10,116.58 vs €11,004.28, *p*-value: 0.001), and neurocognitive impairments events (€7706.43 vs €11,641.29 p- value: < 0.001).

**Conclusions:**

This study provides a differentiated and comprehensive analysis of the healthcare resources needed by HIV and HIV/HCV patients with comorbidities and may contribute to the decision process of resources allocation, in the clinical management of different HIV+ patient populations.

## Background

HIV infection is a lifelong condition, and, as treatment has become more effective, the average age of the people living with HIV (PLWHIV) has increased, with a higher probability of incidence of developing comorbidities, making the clinical and economic management of PLWHIV more complex over time.

Among the major non-AIDS causes of morbidity and mortality in PLWHIV there are [1]: cancers, which comprise both HIV-related and non-HIV related neoplasms [2]; and adverse events due to ART treatments. In particular, hepatotoxicity [3], and renal impairment (present in 5%–32% of HIV+ patients) [1, 4]. Compared with the general population, PLWHIV also have an increased risk of osteoporotic fracture, bone events [5], and comorbidities in general, which require more healthcare services. Neurocognitive and psychiatric disorders are also important in PLWHIV, due to the increased costs of management of HIV disease, in these patients, as a result of lower adherence to ART treatments, and their effects on physical and mental health [6].

HIV/AIDS related or not AIDS-related cancers, are among the major causes of morbidity and mortality for the HIV+ population [2].

Prior economic evaluations in PLWHIV, have been focused on the management expenditure [7, 8]. In fact, authors mainly investigated the different resources requirements of subjects who achieve a virological control, and those who do not [7], with the costs of the antiretroviral drugs available from institutional structures. However, literature evidence concerning the expenditures related to specific comorbidities in PLHIV, are relatively lacking.

In the Italian setting, Quiros-Roldan [8] estimated the direct costs of comorbidities in PLWHIV, compared with the subjects without HIV infection in a province of Lombardy Region (Brescia), and Guaraldi and colleagues [9] conducted a similar analysis in a province of Emilia-Romagna region (Modena).

To date, literature did not provide an accurate and updated economic evaluation of the resource’s requirements for HIV/HCV co-infected patients. Moreover, despite the heterogeneity of the HCV population, the current economic evaluations in the HCV setting provide only an aggregate framework of this pathology [10, 11], focusing especially on the most impacting HCV disease cost: the one related to antiviral therapies.

Therefore, an updated epidemiological and economic analysis of comorbidities in mono-infected HIV+ and co-infected HIV/HCV populations is needed. The present study is aimed at comparing two homogeneous HIV + and HIV/HCV populations (although aware of the potential channeling bias due to their different characteristics), and at analysing the economic resources requirements, according to the number and the type of comorbidities, assuming the Italian National Healthcare Service (NHS) perspective.

## Materials and methods

### Study design

A retrospective observational study was conducted within 3 Public Hospitals in the Lombardy Region, northern Italy (ASST Valle Olona, Busto Arsizio; ASST Fatebenefratelli Sacco, Milano; ASST Santi Paolo e Carlo, Milano).

The study enrolled HIV and HIV/HCV adult patients (age ≥ 18 years), who had been taking ART drugs for at least six months, in charge by Infectious Disease Departments, and who accessed to the services, from January 2016 to December 2016, after receiving the Ethical Committee approval. Enrolled patients presented one or more of the following comorbidities.

- cardiovascular (CDV) events, including hypertension, cardiovascular risk, atrial fibrillation, acute myocardial infarction;
- neurocognitive impairments, including clinically evident HIV-related dementia, cerebral aneurysm;
- HBV infection;
- bone events, including events such as e.g. osteoporosis, osteopenia, bone fractures;
- renal impairments, including calculosis, proteinuria from grade 2 to 3, and kidney failure;
- diabetes.

We also considered a control group composed by mono-infected HIV+ and co-infected HIV/HCV patients, without comorbidity to demonstrate the hypothesis that the economic resources absorption was proportional to the number of comorbidities.

### Data collection

Data derived from an in-depth analysis of administrative databases related to the year 2016 (i.e. clinical or outpatient records of the hospital), including:

- patients’ demographic information (gender and age);
- years of infection (both HIV and HCV);
- comorbidities;
- cost, duration and type of drugs due to the comorbidity;
- cost of hospital admission;
- cost of laboratory tests, diagnostic and specialist procedures needed to diagnose and monitor comorbidities;
- cost, duration and type of antiretroviral treatment;
- cost, duration and type of anti-viral therapy for co-infected HIV/HCV patients.

Economic data were evaluated using outpatients/hospital admission reimbursement tariffs and the Italian NHS official drugs price list, currently valid.

### Statistical methods

A case-control design of the study was structured to guarantee comparability. In particular, each mono-infected HIV+ patient was compared, with a consecutive HIV/HCV co-infected patient, characterized by similar comorbidities.

Data analysis consisted of a comparison between cohorts of mono and co-infected patients considering the following variables: age, gender, years with HIV, years with HCV, number of comorbidities.

Data were analysed considering descriptive statistics, frequencies, and distributions. After having tested the normal distribution of all the variables investigated (− 1 < Skewness< 1 and − 1 < Kurtosis< 1), independent sample t-tests, chi-square test and one-way ANOVAs were used to describe the existence of statistically significant differences between mono-infected and co-infected subjects (considering the same comorbidity or group of comorbidities), from an epidemiological and an economic point of view. All analyses were conducted with a significant level of 0.05.

From an economic perspective, the analysis considered the mean total cost, the mean total cost without HCV therapy cost, the mean total cost without hospitalization/DH events cost and the mean total cost without HCV therapy cost, and hospitalization/DH events cost, comparing the cohorts of mono and co-infected patients.

Moreover, we reported the mean value of HIV therapy cost, laboratory tests cost, diagnostic and specialist procedures cost, drug cost for the two considered cohorts.

The same analysis (from demographical and economical perspectives) was performed for patients with one comorbidity, focusing on the most frequent comorbidities conditions.

In conclusion, a multivariate analysis was performed. A final investigation of the relationships among the variables, using a hierarchical sequential linear regression model (with enter methodology), was implemented to test factors influencing the average management costs, useful in order to establish the impacts of all the comorbidities under assessment.

All statistical analyses were performed using the statistical software SPSS 22.0.

## Results

### Study sample

The sample was composed of 676 patients, 338 suffering from HIV infection and 338 with a concomitant HCV disease.

Although all patients have been enrolled in the Lombardy Region (where HIV+ patients amount to 27,616 (Open Data, Lombardy Region, 2017), the sample could be considered as representative of the entire HIV+ Italian population (equal to 78,000 patients (Centro Operativo AIDS- COA, 2018). The considered sample represented the 2.45% of regional HIV+ population and the 0,87% of the national HIV+ population.

Patients’ characteristics were reported in Table 1. The control group without comorbidities was composed of 338 patients (169 HIV+ and 169 HIV/HCV).

**Table 1:** Detailed information on the sample (patients with and without comorbidities)

	**HIV+ mono-infected patients** ***N* = 507**	**HIV/HCV co-infected patients** ***N* = 507**	***p*-value**
**Gender M [%]**	80.47%	81.06%	> 0.050
**Age [mean ± SD]**	49.00 ± 0.45	51.01 ± 0.24	< 0.001
**Years with HIV [mean ± SD; mode; median]**	12.42 ± 0.33; 11.00;12.00	23.64 ± 0.31; 26.00;29.00	< 0.001
**Years with HCV [mean ± SD; mode; median]**	N.A.	21.15 ± 0,.28; 23.00;23.00	N.A.

Note: N.A. = Not Applicable

The population was composed predominantly of males (81%) for both mono-infected and co-infected subjects, allowing a comparison between these two populations.

There was a statistically significant difference between the mean age of the co-infected population (51 years) and of the mono-infected patients (49 years).

These data were consistent with the general HIV+ and HIV/HCV population taken in charge by the Italian National Healthcare Service [12, 13].

The average duration of HIV infection was 19 years on average, and however, this data varied between the sub-populations: the mono-infected patients were diagnosed with HIV virus more recently than the co-infected subjects, 12.4 years vs. 23.6 years respectively, with an emerging statistically significant difference between the two sub-groups (*p* <  0.001).

In the HIV/HCV co-infected population, the average duration of HCV infection was 21 years. In this population, the HIV virus has firstly been diagnosed, followed by the HCV disease, suggesting that on average, the HIV infected predated HCV infection (Table 1).

Risk factors for HIV+ acquisition were equal distributed in the overall sample (considering both HIV+ and HIV/HCV patients) (p-value: > 0.05): heterosexual (31.4%), homosexual (27.4%), drugs abuse (21%), other (21.2%).

### Comorbidities

When patients were grouped according to the number of comorbidities (Table 2), 251 HIV+ and 200 HIV/HCV patients presented one comorbidity (corresponding with 50% of the mono-infected population and 40% of the co-infected population, chi-squared< 0.001).

**Table 2:** Distribution of the comorbidities among the study populations

	**HIV+ mono-infected patients** ***N*** **= 507**	**HIV/HCV co-infected patients** ***N* = 507**	**Chi-squared**
**No comorbidity**	33.33%	33.33%	> 0.050
**One comorbidity**	49.51%	39.45%	< 0.001
**Two comorbidities**	14.40%	18.54%	> 0.050
**Three or more comorbidities**	2.76%	8.68%	< 0.001

About 9% of the HIV/HCV population suffered from three or more comorbidities (chi-squared< 0.001) (Table 2).

Regarding the two sub-populations with patients with one comorbidity (Table 3), the most frequent comorbidities conditions were cardiovascular, neurocognitive impairments, HBV infection (especially in mono-infected population) and bone events (especially in HIV/HCV subjects).

**Table 3:** Distribution of comorbidities type among the study population with one comorbidity

	**HIV+ patients** ***N*** **= 251**	**HIV/HCV patients** ***N*** **= 200**	**Chi-squared**
**Cardiovascular comorbidities**^a^	164 [65.59%]	84 [42.00%]	< 0.001
**Neurocognitive impairments**^b^	45 [17.99%]	27 [13.50%]	> 0.050
**HBV infection**	25 [10.00%]	9 [4.50%]	0.032
**Bone events**^c^	7 [2.80%]	61 [30.51%]	< 0.001
**Renal impairments**^d^	6 [2.41%]	4 [1.99%]	> 0.050
**Diabetes**	3 [1.20%]	15 [7.50%]	0.001

^a^Cardiovascular (CDV) events, including hypertension, cardiovascular risk, atrial fibrillation, acute myocardial infarction

^b^Neurocognitive impairments, including clinically evident HIV-related dementia, cerebral aneurysm

^c^Bone events, including osteoporosis, osteopenia, arthritis, bone fractures

^d^Renal impairments, including calculosis, proteinuria from grade 2 to 3, and kidney failure

The distribution of comorbidities among the sub-populations with more than one comorbidity is reported in the Additional file 1 section.

The sample with one comorbidity, was composed of patients with the average age was 50 years old; the mono-infected were statistically significantly younger than co-infected patients (49.80 vs 51.67; *p*-value < 0.05) and were predominantly composed of male (about 82%) (Table 4).

**Table 4:** Detailed information analysis of the patients’ sample, with one comorbidity

		**HIV+ mono-infected patients** ***N*** **= 251**	**HIV/HCV co-infected patients** ***N* = 200**	***p*-value and Chi-squared**
**Cardiovascular comorbidity**	Mean Age [y]	53.22	51.80	> 0.050
	Male gender	78.05%	84.52%	> 0.050
	Years with HIV [y]	12.89	23.21	< 0.001
**Neurocognitive impairments**	Mean Age [y]	42.13	50.78	< 0.001
	Male gender	97.78%	85.19%	0.042
	Years with HIV [y]	7.58	21.63	< 0.001
**HBV**	Mean Age [y]	46.40	50.11	> 0.050
	Male gender	100.00%	100.00%	NA
	Years with HIV [y]	18.40	23.00	> 0.050
**Bone events**	Mean Age [y]	43.43	51.31	< 0.001
	Male gender	71.43%	73.77%	> 0.050
	Years with HIV [y]	10.71	24.95	< 0.001
**Renal events**	Mean Age [y]	41.83	53.75	0.014
	Male gender	66.67%	75.00%	> 0.050
	Years with HIV [y]	11.17	20.75	> 0.050
**Diabetes**	Mean Age [y]	44.33	54.40	0.002
	Male gender	100.00%	73.33%	> 0.050
	Years with HIV [y]	14.00	22.93	> 0.050

The average duration of HIV infection in the studied sub-populations stratified considering the different comorbidities was different (Table 4). Please note that the average duration of HIV was equal to 12.37 years for the HIV+ population with one comorbidity and 23.45 for the co-infected HIV/HCV population, with one comorbidity.

### Economic results

On average, the co-infected patients required more resources, in comparison with the mono-infected patients, even excluding the cost of the HCV therapy for the co-infected populations. The average costs increased alongside the number of comorbidities (Table 5).

**Table 5:** Economic impact (mean total cost) of comorbidities, divided by the number of comorbidities

	**HIV+ mono-infected patients**	**HIV/HCV co-infected patients**	**Delta between HIV/HCV and HIV+ patients [%]**	***p*-value between HIV/HCV and HIV+ patients**
**No comorbidity**	€ 8,272.18	€ 12,897.43 € 10,382.67^a^	35.86% 25.51%^a^	< 0.001 < 0.001^a^
**One comorbidity**	€ 9,566.95	€ 17,426.32 € 11,159.68^a^	82.15% 14.27%^a^	< 0.001 0.001^a^
**Two comorbidities**	€ 11,423.22	€ 18,348.50 € 11,896.95^a^	37.74% 3.98%^a^	0.018 > 0.050^a^
**Three or more comorbidities**	€ 12,532.49	€ 12,755.15 € 12,228.53^a^	1.75% − 2.49%^a^	> 0.050 > 0.050^a^
**One way ANOVA**	0.095	0.0554		

^a^Average cost without considering costs incurred for HCV therapy

Regarding the HIV+ population, the mean cost for the subjects without comorbidities was always lower than the one experienced by the sub-population with at least one comorbidity. This result confirmed the hypothesis that the level of resources absorption increased along with the number of developed comorbidities.

The HIV/HCV population showed the same trend, independently from the HCV therapies costs. Only the sub-population with three or more comorbidities displayed a different trend since, in this case, the HCV drug cost category has no significant impact on the patient’s clinical pathway (Table 5).

Regarding the mono-infected subjects with one comorbidity, the economic analysis investigated two specific scenarios.

i) The analysis of total management costs of mono and co-infected patients excluding the cost of HCV drugs; in this scenario, HIV/HCV patients showed a higher resources absorption than mono-infected HIV+ patients (about + 14%).
ii) The analysis of the year 2016 excluding episodic event of hospitalization, not always related to the specific comorbidity or principal diagnosis, but to other accidental factors occurred to the patient (a fracture, for example, not related to the bone density, or other accidental eventuality).

The results demonstrated that co-infected subjects required a higher mean total cost resource use, than mono-infected patients (+ 50%) (see Additional file 1 for the sample presenting more than one comorbidity).

Table 6 reports the different economic scenarios, comparing the two sub-populations without the costs of HCV therapy and day hospital/hospitalization events (co-infected population absorbed + 22% of resources).

**Table 6:** Economic impact of HIV and HIV/HCV populations with one comorbidity

	**HIV+ mono-infected patients**	**HIV/HCV** **co-infected patients**	**Delta between HIV/HCV and HIV+ patients [%]**	***p*-value**
**Mean total cost**	€ 9,566.95	€ 17,426.32	45.10%	< 0.001
**Mean total cost without HCV therapy cost**	€ 9,566.95	€ 11,159.68	14.27%	0.001
**Mean total cost without hospitalization/DH events cost**	€ 8,759.71	€ 17,425.76	49.73%	< 0.001
**Mean total cost without HCV therapy cost and hospitalization/DH events cost**	€ 8,759.71	€ 11,159.12	21.50%	< 0.001
**Mean HIV therapy cost**	€ 7,117.33	€ 9845.34	27.71%	< 0.001
**Mean laboratory tests cost**	€ 880.19	€ 468.45	−87.89%	0.001
**Mean diagnostic and specialist procedures cost**	€ 452.52	€ 204.58	− 121.20%	< 0.001
**Mean drug cost**	€ 309.68	€ 640.74	51.67%	< 0.001

**Fig. 1:**
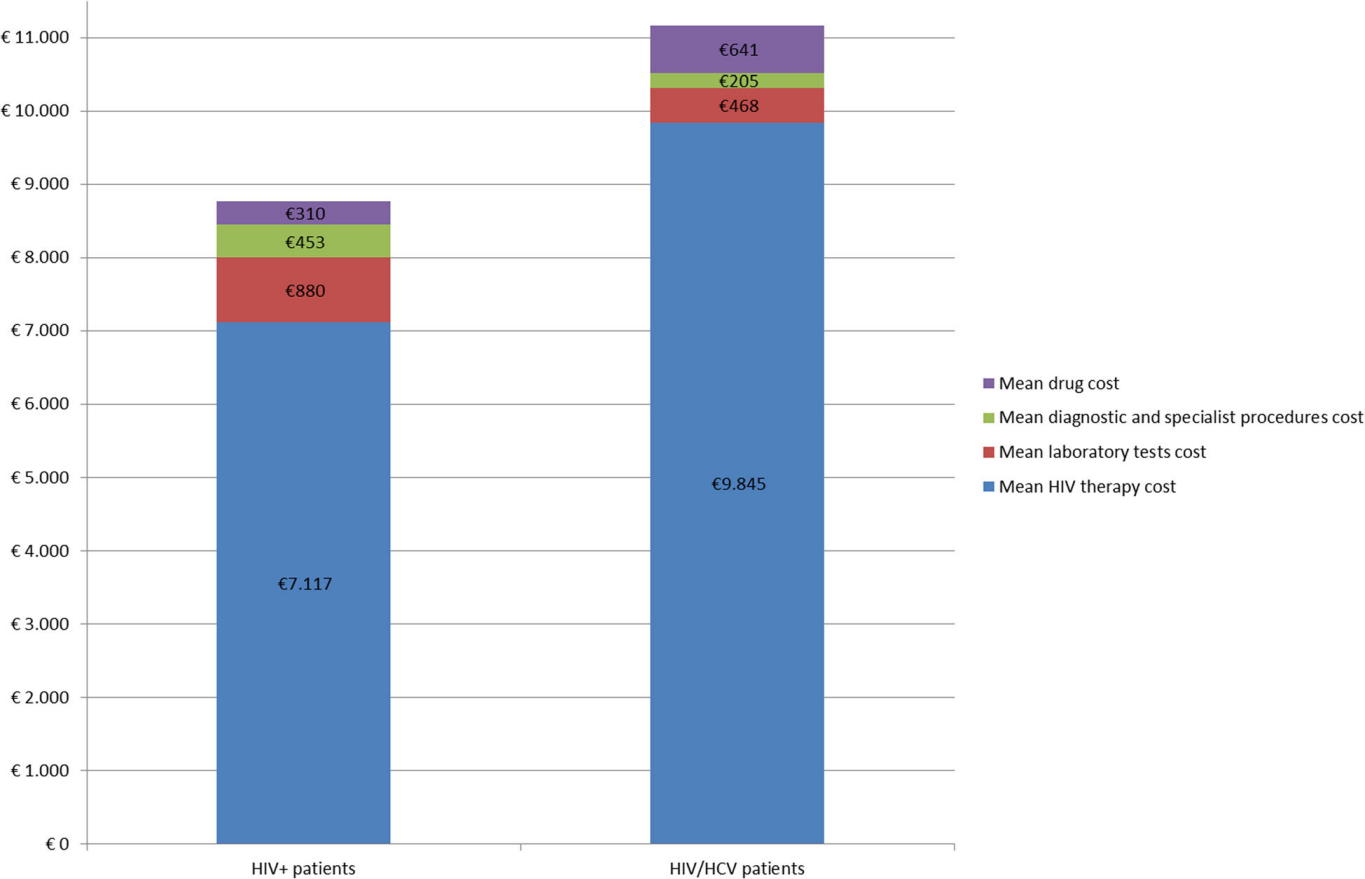
Economic impact (cost items), excluding the related costs for HCV therapy, in HIV+ and HIV/HCV populations, with one comorbidity

The average costs divided per cost item (HIV therapy, laboratory tests, diagnostic and specialistic procedures, drugs prescribed for the comorbidity) were also analysed (Table 6, Fig. 1). HIV/HCV subjects showed a higher cost for HIV therapy and other drugs.

Taking into consideration all the cost items and the performed scenarios, statistically significant differences emerged between sub-populations.

**Fig. 2:**
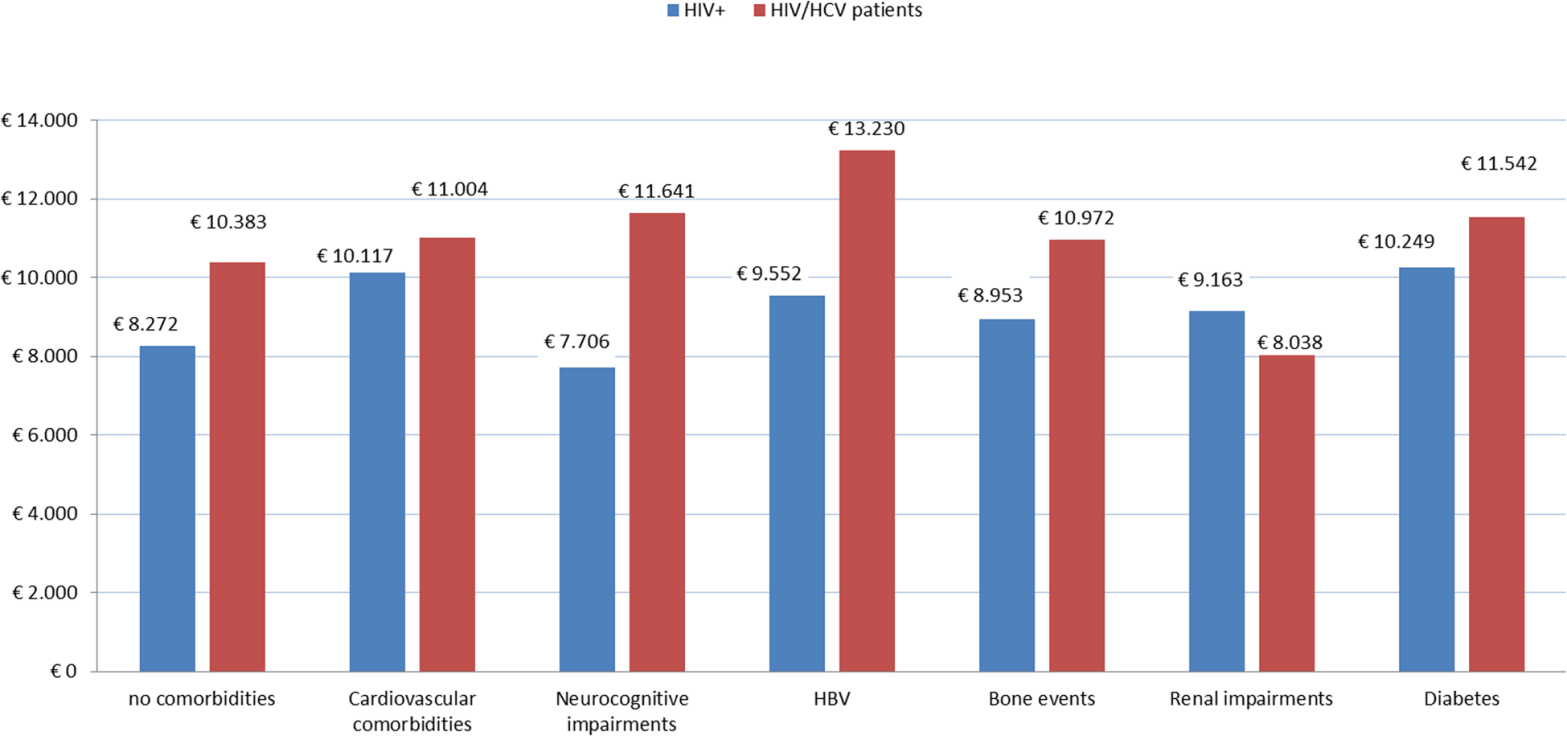
Economic impact of observed comorbidities (mean total cost) among the populations with one comorbidity

Regarding the mono-infected subjects with one comorbidity, patients with cardiovascular comorbidities and diabetes absorbed more resources, whereas the co-infected population with HBV, diabetes and cardiovascular comorbidity registered a higher mean total cost (Table 7; Fig. 2; see Additional file 1 for the sample with more than one comorbidity).

**Table 7:** Economic impact of observed comorbidities (mean total cost) among the populations with one comorbidity. (average cost excluding the related HCV therapy costs)

	**HIV+** **Mono-infected patients** ***N*** **= 251**	**HIV/HCV** **Co-infected patients** ***N* = 200**	**Delta between HIV/HCV and HIV+ patients** [%]	***p*-value between HIV/HCV and HIV+ patients**
**Cardiovascular comorbidities**	€ 10,116.58	€ 20,724.95 € 11,004.28*	51.19% 8.07%*	0.001 > 0.050*
**Neurocognitive impairments**	€ 7,706.43	€ 11,641.29 € 11,641.29*	33.80% 33.80%*	< 0.001 < 0.001*
**HBV infection**	€ 9,551.84	€ 28,790.25 € 13,230.36*	66.82% 27.80%*	> 0.050 > 0.050*
**Bone events**	€ 8,953.19	€ 12,097.29 € 10,972.37*	25.99% 18.40%*	> 0.050 > 0.050*
**Renal impairments**	€ 9,162.64	€ 8038.49 € 8038.49*	−13.98% − 13.98%*	> 0.050 > 0.050*
**Diabetes**	€ 10,249.13	€ 26,723.49 € 11,541.70*	61.65% 10.99%*	> 0.050 > 0.050*
**One-way ANOVA**	0.215	0.017		

*Average cost without considering costs incurred for HCV therapy

Analysing the economic impact (mean total cost) in terms of differences across sub-groups of comorbidities and focusing on the entire HIV mono-infected sample (not considering the number of comorbidities), higher resources absorption emerged with regard to the presence of cardiovascular diseases (*p*-value = 0.009), bone diseases (p-value = 0.028) and diabetes (*p*-value = 0.029) (Table 8). The same trend emerged within the HIV/HCV co-infected population, where patients suffering from HBV infection presented higher costs for their management (*p*-value =0.035) (Table 8).

**Table 8:** Economic impact (mean total cost): differences across sub-groups of comorbidities

	**The entire sample**			**HIV mono-infected patients**			**HIV/HCV co-infected patients**		
**HCV Infection**	**Average Value**	**Standard Error**	***p*-value**	**Average Value**	**Standard Error**	***p*-value**	**Average Value**	**Standard Error**	***p*-value**
Absence of HCV infection	10,090.69 €	334.75 €	**0.000**	*Not Applicable*	*Not Applicable*	*Not Applicable*	*Not Applicable*	*Not Applicable*	*Not Applicable*
Presence of HCV infection	17,074.70 €	1,187.14 €		*Not Applicable*	*Not Applicable*		*Not Applicable*	*Not Applicable*	
**Cardiovascular disease**	**Average Value**	**Standard Error**	***p*-value**	**Average Value**	**Standard Error**	***p*-value**	**Average Value**	**Standard Error**	***p*-value**
Absence of cardiovascular disease	12,070.49 €	792.10 €	**0.048**	8,717.72 €	442.80 €	**0.009**	14,377.01 €	1,267.71 €	**0.037**
Presence of cardiovascular disease	14,404.40 €	871.29 €		10,643.30 €	429.75 €		19,005.54 €	1,813.91 €	
**Neurocognitive impairment**	**Average Value**	**Standard Error**	***p*-value**	**Average Value**	**Standard Error**	***p*-value**	**Average Value**	**Standard Error**	***p*-value**
Absence of neurocognitive impairment	14,518.94 €	800.07 €	**0.000**	10,425.92 €	395.75 €	**0.042**	18,884.83 €	1,554.33 €	**0.000**
Presence of neurocognitive impairment	10,271.29 €	301.04 €		8,709.13 €	497.84 €		11,513.49 €	308.11 €	
**HBV Infection**	**Average Value**	**Standard Error**	***p*-value**	**Average Value**	**Standard Error**	***p*-value**	**Average Value**	**Standard Error**	***p*-value**
Absence of HBV infection	13,164.39 €	643.60 €	0.153	10,121.96 €	376.05 €	0.831	15,960.67 €	1,164.25 €	**0.035**
Presence of HBV infection	16,571.34 €	2,276.63 €		9,926.26 €	698.56 €		28,944.94 €	5,772.21 €	
**Bone disease**	**Average Value**	**Standard Error**	***p*-value**	**Average Value**	**Standard Error**	***p*-value**	**Average Value**	**Standard Error**	**p-value**
Absence of bone disease	14,089.24 €	83.74 €	**0.043**	9,878.89 €	316.44 €	**0.028**	21,076.62 €	2,064.49 €	**0.000**
Presence of bone disease	12,143.66 €	469.12 €		12,632.31 €	2,099.83 €		12,058.96 €	417.99 €	
**Renal Impairment**	**Average Value**	**Standard Error**	***p*-value**	**Average Value**	**Standard Error**	***p*-value**	**Average Value**	**Standard Error**	***p*-value**
Absence of renal impairment	13,749.50 €	666.56 €	**0.012**	10,099.74 €	352.08 €	0.922	17,297.56 €	1,238.65 €	0.384
Presence of renal impairment	10,858.28 €	909.77 €		9,972.35 €	1,025.99 €		12,275.78 €	1,685.48 €	
**Diabetes**	**Average Value**	**Standard Error**	***p*-value**	**Average Value**	**Standard Error**	***p*-value**	**Average Value**	**Standard Error**	***p*-value**
Absence of diabetes	12,859.43 €	577.95 €	**0.045**	9,884.66 €	340.45 €	**0.029**	16,137.97 €	1,124.92 €	**0.035**
Presence of diabetes	19,048.37 €	3,120.83 €		12,670.23 €	1,452.30 €		22,001.21 €	4,472.99 €	

For both groups of patients, presenting neurocognitive impairments did not lead to a greater resources’ absorption (*p*-value ≤0.005) (Table 8).

A regression analysis was conducted, thus examining the Adjusted R2, in order to control the explanatory power of the types of comorbidity in relation to each investigated population (Table 9).

**Table 9:** Regression model: mean total cost as dependent variable and types of comorbidity as independent variables

	Mean total cost Entire Sample		Mean total cost HIV+ monoinfected patients		Mean total cost HIV/HCV coinfected patients	
Independent variables	Beta	Sig.	Beta	Sig.	Beta	Sig.
Cardiovascular comorbidities	0.268	0.007	0.156	0.000	0.178	0.001
Neurocognitive impairments	−0.124	0.214	−0.029	0.467	−0.016	0.762
HBV infection	0.074	0.123	0.029	0.874	0.092	0.041
Bone events	−0.236	0.000	0.545	0.000	−0.036	0.497
Renal impairments	−0.112	0.003	−0.113	0.529	−0.010	0.850
Diabetes	0.233	0.000	0.488	0.000	0.449	0.000
R2	0.191		0.620		0.275	
Adjusted R2	0.180		0.610		0.259	
F value	17.794		67.719		17.243	
Δ R2	0.191		0.620		0.275	
F (ΔR2)	17.794		67.719		17.243	

The regression analysis confirmed the previous results concerning the differences across sub-groups of comorbidities. In particular, predictors of mean total cost in the sample were the presence of cardiovascular comorbidities (β = 0.268; *p* = 0.007) and diabetes (β = 0.233; p = 0.000) and the absence of bone events (β = − 0.236; *p* = 0.000) and renal impairments (β = − 0.112; *p* = 0.003). These predictors were confirmed also in the HIV+ monoinfected population, except for renal impairments. Cardiovascular comorbidities and diabetes were the main predictors in the HIV/HCV coinfected population (Table 9).

## Discussion

This study contributes to defining the costs’ patterns associated with HIV+ and HCV/HIV patients. This study provides accurate and detailed estimates of the annual economic impact of the HIV+ population on healthcare expenditure, to support the planning and the decision making of policy makers.

Recent guidelines, such as the Italian and Australian ones, have addressed the issues of comorbidities related to antiretroviral therapy for HIV [14, 15]. Moreover, these guidelines encompass a tailored and multidisciplinary approach to HIV+ patients. Therefore, a multidisciplinary approach to patient management and to the clinical pathway of HIV+ patients, coordinated by the Infectious Disease Specialists, may lead to a significant improvement in the healthcare services. In this view, the design and implementation of a structured patient’s pathway, are desirable to standardize the process of taking in charge, caring and monitoring the HIV+ populations with comorbidities, and to reduce the differences in resources absorption.

In contrast to current literature [7, 16, 17], which is focused on the expenditure cost of antiretroviral or the anti-hepatitis antiviral drugs, or the management cost of the HIV+ subjects excluding comorbidities, our study provides a more comprehensive analysis, encompassing the multidisciplinary views for the management of HIV+ patients and their comorbidities.

The comorbidities’ cost has been analysed by few previous studies, that considered only the mono-infected population. Guaraldi [9] and colleagues demonstrated that the direct costs of medical care, including the coverage of comorbidities ones, was higher for HIV+ patients than for uninfected subjects, stratified by age, given the ART costs. Quiros-Roldan [8] and colleagues estimated the cost of HIV+ infected patients with chronic diseases, emphasizing the high burden of HIV infection in terms of costly and emerging comorbidities such as diabetes, cardiovascular disease, neoplasms, and dyslipidaemia.

In our study, the co-infected patients, on average, required more resources than the mono-infected subjects irrespective of the type of comorbidity. This data could be ascribed to the fact that, the HIV/HCV patients involved, showed significant differences compared with HIV+ patients. For example, co-infected patients presented had more years of exposure to HIV infection, and a longer experience of antiretroviral therapy. Our results suggested that, in the co-infected population, the mean total cost is due to the type of comorbidity alongside the severity of hepatitis C. The coexistence of these infections worsens the clinical status of the patient and, consequently, increases the absorption of healthcare resources. Moreover, in the co-infected population, the mean total cost is also determined by the HCV therapy since in 2014–2015 the landscape of anti-HCV medications has rapidly evolved introducing several more effective and expensive alternative technologies, even not comparable with the actual use of DAAs.

The comparative analysis between mono-infected and co-infected populations has highlighted significant differences that may explain the dissimilar cost-absorption, not proportional to comorbidities and stratification. Furthermore, previous evidence proved that HIV infection could accelerate the progression to cirrhosis increasing the viral replication ability and quickening the development of the hepatic disease [15], with a consequent increase of fibrosis progression and mortality associated with HCV.

Cardiovascular comorbidities, neurocognitive disorders, HBV infection and bone events represent the most frequent comorbidities in the populations of our study. The early management of these comorbidities could have a positive impact not only from a clinical point of view, but also from an economic perspective.

The results proposed in the present study, were consistent with the existing literature evidence. In the Swiss HIV Cohort Study, the role of cardiovascular disease, osteoporosis, diabetes mellitus, and non–AIDS-defining malignancies, have been become ever more relevant in HIV-infected persons, and increasing with the patients’ ageing. Our data suggest that, if the above-mentioned comorbidities are incurred in co-infected patients, the absorption of economic resources might increase; hence, reducing or delaying the development of comorbidities in HIV and HIV/HCV patients may save resources.

The main strength of the present study is the “real-life” setting of the analysis that overviews all the healthcare items of expenditure related to the management of HIV and HIV/HCV subjects who develop comorbidities, thus providing a reliable estimation of costs.

A limitation of the present study is the sample size, since a limited number of subjects with specific comorbidities were enrolled. This aspect limited the statistical power of the comparison between HIV and HIV/HCV patients and made it difficult to compare the two populations when two or more comorbidities were present. However, the sample of the study and the data concerning patients with one comorbidity were robust, thus confirming the usefulness of the achieved information.

## Conclusion

In conclusion, the present study provides a differentiated and comprehensive pattern of the healthcare resources needed by HIV+ and HIV/HCV patients with comorbidities and may contribute to the decision process of resources allocation, in the clinical management of different HIV+ populations in Italy.

Future researches may be planned to update the data of the present study, in order to monitor the trend of the comorbidities over time, and thus provide a longitudinal clinical and economic perspective.

Moreover, the results of this study may pave the way to implement specific guidelines related to the management of the comorbidities of HIV+ patients by the infectious disease specialists.

## Supplementary information

**Additional file 1.**

## Data Availability

The datasets used and/or analysed during the current study are available from the corresponding author on reasonable request.

## Abbrevations

AIDS: Acquired Immune Deficiency Syndrome
ANOVA: ANalysis Of VAriance
ART: Antiretroviral Therapy
CDV: Cardiovascular
DAA: Direct-Acting Antiviral
DH: Day Hospital
HBV: Hepatitis C Virus
HCV: Hepatitis C Virus
HIV: Human Immunodeficiency Virus
PLWHIV: People living with HIV
SPSS: Statistical Package for Social Sciences
