# Supplementary material for: Comorbidities and HCV coinfection in the management of HIV+ patients: evidence from the Italian clinical practice
Source: Health Econ Rev. 2020 Aug 29;10:27. doi: 10.1186/s13561-020-00284-x (PMC7456501; doi:10.1186/s13561-020-00284-x)
Supplement: Supplementary file 1 — Additional file 1. [file 13561_2020_284_MOESM1_ESM.docx]

**Distribution of comorbidities type among the study population presenting two comorbidities.**

|  | **HIV+ patients**  **N=338** | **HIV/HCV patients**  **N=338** |
| --- | --- | --- |
| **CDV+Diabetes** | 5.03% | 4.73% |
| **CDV+Bone events** | 4.44% | 9.47% |
| **HBV+CDV** | 4.14% | 2.07% |
| **CDV+Renal impairments** | 3.25% | 0.89% |
| **HBV+Neurocognitive impariments** | 2.66% | 0.59% |
| **CDV+Neurocognitive impariments** | 1.48% | 3.55% |
| **CDV+Cancer** | 0.59% |  |
| **HBV+Diabetes** |  | 0.59% |
| **HBV+Bone events** |  | 1.18% |
| **BONE+Diabetes** |  | 0.59% |
| **BONE+Renal impairments** |  | 0.30% |
| **Neurocognitive impariments+Diabetes** |  | 0.30% |
| **Neurocognitive impariments+Bone events** |  | 3.55% |

**Economic impact of HIV and HIV/HCV populations with two comorbidities.**

|  | **HIV+ patients** | **HIV/HCV patients** | **Delta %** | **p-value** |
| --- | --- | --- | --- | --- |
| **Mean total cost** | € 11,423.22 | € 18,348.50 | 37.74% | 0.018 |
| **Mean total cost without HCV therapy cost** | € 11,423.22 | € 11,896.95 | 3.98% | >0.050 |
| **Mean total cost without hospitalization/DH events cost** | € 10,554.64 | € 18,300.10 | 42.32% | 0.008 |
| **Mean total cost without HCV therapy cost and hospitalization/DH events cost** | € 10,554.64 | € 11,848.56 | 10.92% | >0.050 |
| **Mean HIV therapy cost** | € 7,891.32 | € 10,269.93 | 23.16% | <0.001 |
| **Mean laboratory tests cost** | € 902.24 | € 483.62 | -86.56% | 0.003 |
| **Mean diagnostic and specialist procedures cost** | € 461.97 | € 213.22 | -116.67% | 0.001 |
| **Mean drug cost** | € 1,299.12 | € 881.79 | -47.33% | >0.050 |

**Economic impact of observed comorbidities (mean total cost) among the population presenting two comorbidity.**

|  | **HIV+ patients** | **HIV/HCV patients** | **Delta %** | **p-value** |
| --- | --- | --- | --- | --- |
| **CDV+Diabetes** | € 12,303.03 | € 28,985.61 | 57.55% | >0.050 |
| **CDV+Bone events** | € 14,449.30 | € 11,826.58 | -22.18% | >0.050 |
| **HBV+CDV** | € 10,225.45 | € 60,850.81 | 83.20% | 0.009 |
| **CDV+Renal impairments** | € 8,199.30 | € 13,905.81 | 41.04% | 0.033 |
| **HBV+ Neurocognitive impariments** | € 9,778.78 | € 11,627.02 | 15.90% | >0.050 |
| **CDV+ Neurocognitive impariments** | € 11,711.05 | € 11,663.84 | -0.40% | >0.050 |
| **CDV+Cancer** | € 14,045.44 |  | NA | NA |
| **HBV+Diabetes** |  | € 12,679.31 | NA | NA |
| **HBV+Bone events** |  | € 11,752.11 | NA | NA |
| **BONE+Diabetes** |  | € 15,978.93 | NA | NA |
| **Bone events+Renal impairments** |  | € 9,761.02 | NA | NA |
| **Neurocognitive impariments+Diabetes** |  | € 10,416.08 | NA | NA |
| **Neurocognitive impariments+Bone events** |  | € 10,595.29 | NA | NA |

Note: N.A.= Not Applicable

**Distribution of comorbidities type among the study population presenting three or more comorbidities.**

|  | **HIV+ patients**  **N=338** | **HIV/HCV patients**  **N=338** |
| --- | --- | --- |
| **HBV+CDV+Neurocognitive impairments** | 1.18% |  |
| **HBV+CDV+Renal impairments** | 0.59% |  |
| **CDV+Diabetes+Cancer** | 0.30% |  |
| **CDV+Diabetes+Renal impairments** | 0.30% | 1.18% |
| **CDV+Bone events+Diabetes** | 0.30% | 1.48% |
| **CDV+Renal impairments +Cancer** | 0.30% | 0.00% |
| **CDV+Neurocognitive impairments+Diabetes** | 0.30% | 0.30% |
| **Neurocognitive impairments+Bone events+Renal impairments** | 0.30% |  |
| **CDV+Bone events+Renal impairments** |  | 0.30% |
| **CDV+Neurocognitive impairments+Bone events** |  | 6.21% |
| **HBV+CDV+Bone events** |  | 0.59% |
| **HBV+Neurocognitive impairments+Bone events** |  | 0.30% |
| **CDV+Bone events+Diabetes+Renal impairments+Cancer** | 0.30% |  |
| **CDV+Neurocognitive impairments+Bone events+Renal impairments** | 0.30% |  |
| **CDV+Bone events+Diabetes+Renal impairments** |  | 0.59% |
| **CDV+Neurocognitive impairments +Bone events+Diabetes** |  | 1.48% |
| **HBV+CDV+Diabetes+Cancer** |  | 0.30% |
| **HBV+CDV+Neurocognitive impairments +Bone events** |  | 0.30% |

**Economic impact of HIV and HIV/HCV populations with three or more comorbidities.**

|  | **HIV+ patients** | **HIV/HCV patients** | **Delta %** | **p-value** |
| --- | --- | --- | --- | --- |
| **Mean total cost** | € 12,532.49 | € 12,755.15 | 1.75% | >0.050 |
| **Mean total cost without HCV therapy cost** | € 12,532.49 | € 12,228.53 | -2.49% | >0.050 |
| **Mean total cost without hospitalization/DH events cost** | € 11,635.18 | € 12,755.15 | 8.78% | >0.050 |
| **Mean total cost without HCV therapy cost and hospitalization/DH events cost** | € 11,635.18 | € 12,228.53 | 4.85% | >0.050 |
| **Mean HIV therapy cost** | € 8,562.23 | € 10,037.97 | 14.70% | >0.050 |
| **Mean laboratory tests cost** | € 858.81 | € 483.75 | -77.53% | >0.050 |
| **Mean diagnostic and specialist procedures cost** | € 464.60 | € 246.37 | -88.58% | >0.050 |
| **Mean drug cost** | € 1,749.53 | € 1,460.44 | -19.80% | >0.050 |

**Economic impact of observed comorbidities (mean total cost) among the population presenting three or more comorbidities.**

|  | **HIV+ patients** | **HIV/HCV patients** | **Delta %** | **p-value** |
| --- | --- | --- | --- | --- |
| **HBV+CDV+Neurocognitive impairments** | € 11,414.20 |  | NA | NA |
| **HBV+CDV+Renal impairments** | € 10,200.00 |  | NA | NA |
| **CDV+Diabetes+Cancer** | € 12,004.42 |  | NA | NA |
| **CDV+Diabetes+Renal impairments** | € 28,187.35 | € 15,738.40 | -79.10% | >0.050 |
| **CDV+Bone events+Diabetes** | € 11,911.03 | € 16,440.89 | 27.55% | >0.050 |
| **CDV+Renal impairments +Cancer** | € 8,463.66 |  | NA | NA |
| **CDV+Neurocognitive impairments +Diabetes** | € 11,714.41 | € 12,913.69 | 9.29% | >0.050 |
| **Neurocognitive impairments+Bone events+Renal impairments** | € 15,425.78 |  | NA | NA |
| **CDV+Bone events +Renal impairments** |  | € 13,449.82 | NA | NA |
| **CDV+Neurocognitive impairments +Bone events** |  | € 11,529.65 | NA | NA |
| **HBV+CDV+Bone events** |  | € 14,610.97 | NA | NA |
| **HBV+Neurocognitive impairments +Bone events** |  | € 7,890.15 | NA | NA |
| **CDV+Bone events+Diabetes+Renal impairments +Cancer** | € 13,039.61 |  | NA | NA |
| **CDV+Neurocognitive impairments +Bone events+Renal impairments** | € 8,651.81 |  | NA | NA |
| **CDV+Bone events+Diabetes+Renal impairments** |  | € 12,050.41 | NA | NA |
| **CDV+Neurocognitive impairments +Bone events+Diabetes** |  | € 12,953.45 | NA | NA |
| **HBV+CDV+Diabetes+Cancer** |  | € 8,770.98 | NA | NA |
| **HBV+CDV+Neurocognitive impairments +Bone events** |  | € 12,831.17 | NA | NA |

Note: N.A.= Not Applicable
